# Superior Vena Cava Syndrome: A Palliative Approach to Treatment

**DOI:** 10.7759/cureus.27889

**Published:** 2022-08-11

**Authors:** Kathryn D Esposito, Masood A Shariff, Aubrey Freiberg, Ma. Carla Angela Evangelista

**Affiliations:** 1 Internal Medicine, St. George's University School of Medicine, St. George's, GRD; 2 Internal Medicine, NYC Health + Hospitals/Lincoln, Bronx, USA; 3 Radiation Oncology, NYC Health + Hospitals/Lincoln, Bronx, USA; 4 Internal Medicine, NYC Health + Hospitals/Lincoln, Lincoln Medical Center, Bronx, USA

**Keywords:** oncologic emergency, refusal of care, patient rights, superior vena cava syndrome, palliative radiation

## Abstract

Superior vena cava (SVC) syndrome is an oncologic emergency of venous congestion due to impaired venous flow through the SVC to the right atrium, leading to potential hemodynamic instability. We report a case of a 78-year-old female patient with a non-symptomatic lung nodule that exhibited rapid growth from its discovery to an enlarging tumor impinging the SVC in less than one month. The short time span from computed tomography (CT) image of the tumor to oncologic emergency required our team to act quickly to identify the source of the tumor and halt its progression, utilizing a multidisciplinary team approach while dealing with a patient that executed their right of autonomy to refusal of care, thus focusing on management with palliative goals since SVC syndrome has a life expectancy of six months post-diagnosis.

## Introduction

Superior vena cava (SVC) syndrome is an oncologic emergency of both malignant and benign etiologies. Patients exhibit hemodynamic instability, congestion, and neurological symptoms.

Most known SVC syndrome cases are malignancy-driven; non-small cell lung cancer (22-57%) and small cell lung cancer (10-39%) comprise the highest prevalence of the cases due to underlying malignancy [1]. Most patients will exhibit face and neck swelling as one of the key identifiers. Previously, dilated chest collateral veins were believed to be one of the hallmarks of the disease, but in clinical practice, it is only seen in about 30% of cases [1]. Treatment is targeted at the underlying etiology; chemotherapy with or without adjuvant radiation is the gold standard of care for malignant cases [1]. SVC syndrome generally requires prompt recognition and intervention to avoid rapid decompensation.

We are reporting a case of a 78-year-old woman with prior oncological history who was diagnosed with a lung nodule one month prior to her presenting in distress from likely compression of the SVC by the tumor. The pathology of this lung nodule remained undetermined, and the patient did not want invasive procedures or therapies as part of her treatment plan. This case presents a patient that adamantly requested non-invasive procedures for an etiology that required an invasive treatment. With the use of palliative radiation, the patient was discharged home with marked relief of respiratory distress compared to her initial presentation. Prompt recognition and urgent radiation treatment while respecting the patient’s wishes for no additional biopsies, surgery, or invasive procedures were pivotal for keeping this patient stable.

## Case presentation

A 78-year-old female presented to our emergency department (ED) on July 13, 2021, in respiratory distress with worsening shortness of breath and apparent use of accessory muscles for breathing. Her oxygen saturation in room air was 80% and increased to 88-90% on the nasal cannula. She was then switched to bilevel positive airway pressure (BiPAP) with head elevation, which resulted in greater than 96% SpO_2_. The patient denied associated fever, chills, cough, chest, and abdominal pain. The patient was tachypneic and tachycardiac. The primary team advised the need for intubation, but the patient refused. Superficial veins of the left anterior chest were dilated with a working diagnosis of SVC syndrome. The patient’s past medical history is comprised of hypertension, asthma/emphysema, heart failure with reduced ejection fraction (HFrEF) with a 33% ejection fraction, hyperlipidemia, osteoporosis, and an unspecified lung nodule diagnosed in early 2019. The lung nodule was of unknown etiology. It had been further evaluated at another facility by computed tomography (CT)-scan and bronchoscopy with ultrasound-guided sampling, and both studies were non-diagnostic. 

In early 2019, she had an evaluation for an enlarged right upper lobe speculated lung nodule for which she had a CT-guided lung node biopsy performed, and the pathology at that time was non-diagnostic. The patient thereafter had multiple negative biopsies with no conclusive diagnosis. At that point, the patient decided not to undergo invasive procedures, and the family respected her wishes. A follow-up CT-scan five months later showed a stable lung lesion compared to the prior study, so no further workup was recommended.

**Figure 1:**
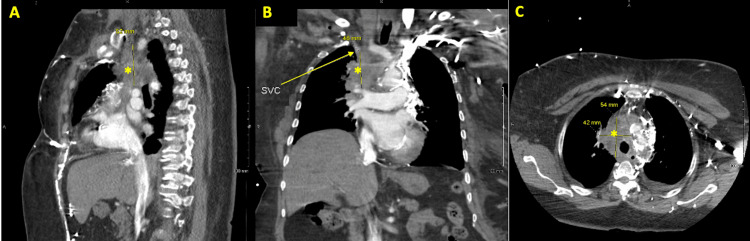
Chest computed tomography. Chest computed tomography (CT) scan shows 54 mm lymphoid mass in the mediastinum (*, asterisk) sagittal view (A). On a coronal image, the mass (*) is extended to the right atrium and compressing the superior vena cava (SVC, arrow) and right brachiocephalic vein (arrow) (B). Transverse lung window CT image shows the mass (*, 54 mm × 42 mm) fully occupying and compressing the SVC (not shown) (C). The vessels on the left side of the mediastinum are opacified, likely related to SVC obstruction.

**Figure 2:**
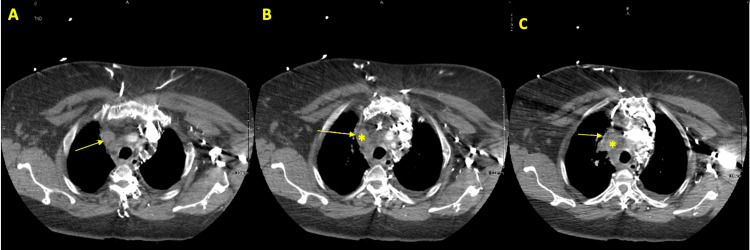
Transverse soft tissue CT slices. Transverse soft tissue CT slices showing the progressive compression of the SVC (arrow) by the mass (*, asterisk) (A->B->C). SVC: superior vena cava. CT: computed tomography.

Imaging was repeated in May 2021, showing a larger nodule increased in size as well as mediastinal lymphadenopathy (Figure 1, 2). 

The next step in the patient’s workup would have been surgery. However, the patient was firm in her decision to have no invasive diagnostic procedures, and the thoracic and oncologic surgery teams did not get involved. On presentation, a CT scan showed worsening mediastinal lymphadenopathy with encasing of the SVC. The patient was admitted to a different facility and was diagnosed with a possible SVC syndrome. There, she underwent another endobronchial ultrasound (EBUS) bronchoscopy, which came back non-diagnostic yet again. A final EBUS bronchoscopy was conducted one week later (June 2021). At that time, the patient once again refused further interventions. However, she was agreeable to CT and EBUS as these were less invasive, and she knew what to expect with the procedures from her previous experience. The EBUS revealed a poorly differentiated carcinoma of unknown origin. At that facility, the patient required oxygen supplementation for her hypoxia, and she was discharged home with supplemental oxygen.

Then the patient presented to our facility three weeks later, on July 13, 2021, to the ED in respiratory distress with worsening shortness of breath. She refused intubation, which was indicated to maintain her oxygen saturation. Instead, she was placed on BiPAP. The family presented to the bedside and said they wanted to follow their mother’s wishes for no intervention. The refusal of treatment was documented, and conservative management was sought. On the same day of ED admission, hematology-oncology and radiation oncology were consulted, and palliative care was on board. A review of pathology reports and slides demonstrated scant malignant spindled and epithelioid neoplasm with a sclerotic background. Immunostaining was positive for cytokeratin AE/AE3, CAM 5.2, vimentin, and cytokeratin and negative for CDX2, TTF1, and p40, indicative of a poorly differentiated carcinoma with spindle features. No evidence was found of this lesion possibly being metastatic. Conversation with the prior facility determined that the obtained tissue sample was insufficient for molecular testing or PDL1 staining. The imaging was re-reviewed for a primary source for the tumor, but none was found. Based on her current condition of hypoxia and HFrEF of 33%, oncologists deemed chemotherapy or immunotherapy an unfit choice for the patient.

After discussion with the patient and family over video conference, an expedited decision to utilize palliative external beam radiotherapy toward the encasing mass over the SVC was scheduled as the family respected the mother’s wishes for no more invasive diagnostic or therapeutic treatments. The goal of palliative radiation was 10 daily sessions at 300 cGy-a total of 3000 cGy-to the area of the mass and SVC compression, which was completed during the patient’s hospital course. Steroids were added to her medication regime after the completion of five sessions of radiation. There was a marked reduction in swelling of the extremities and face by day five. The primary team scheduled a second CT scan, but the patient did not agree to get it done. The patient still endorsed shortness of breath with continued BiPAP use at night and a high-flow nasal cannula or nasal cannula only during the day (Table 1). 

**Table 1:** Trend of radiation therapy treatments and oxygen requirements. Demonstrates the trend of radiation therapy treatments enabling better oxygenation and lessened work of breathing. The patient was able to go from BiPAP dependence to nasal cannula after radiation therapy.

Hospital day	Treatment	Oxygen therapy interfaces	SpO_2_
1	Admission	Room air	80%
	ED	Nasal cannula	88-90%
	First radiation session	BiPAP (12/6, RR 14/40%)	96%
8	Sixth radiation session	HFNC 40/40	92%
14	Tenth (final) radiation session	HFNC 40/40	87-95%
17	Awaiting discharge	Nasal cannula-4L	94%
18 (Discharge day)	Discharged-portable/home oxygen	Nasal cannula-5L	93%
ED: emergency department; BiPAP: bilevel positive airway pressure; HFNC: high-flow nasal cannula; Spo_2_: blood oxygen saturation level; RR: respiratory rate.			

It is likely that the tumor size decreased due to improvement in the patient's respiratory symptoms, but because the patient refused further CT scans, a definitive size decrease remains unknown. A family meeting with the multidisciplinary teams was conducted to decide the patient’s goals of care, and the patient and family agreed to opt for home hospice. The patient was discharged with home hospice, oxygen at 5L nasal cannula, and a low dose of alprazolam to reduce the subjective work of breathing to help her feel better.

## Discussion

In patients presenting with SVC syndrome due to malignancy as opposed to benign etiology, establishing a definitive pathological diagnosis with microscopic evaluation and immunohistochemistry of the malignancy aids in choosing a specific treatment and determining the urgency of medical intervention. The patient’s EBUS with biopsies yielded scant samples making immunohistochemistry inconclusive; primary pathology could not be determined. In addition, repeat imaging did not show clear evidence of a primary mass. The team could not offer immunotherapy to the patient due to inconclusive PDL1 staining with the available samples. Offering the patient a definitive treatment was challenging for both the oncology and primary teams, given the patient’s fragile condition and the unclear benefits of non-palliative treatment. In this patient, the primary pathology of the lung nodule causing SVC syndrome remained unknown.

Radiation oncologists recommend that diagnostic procedures for tissue diagnosis are done prior to radiation; after a course of radiation, the tissue biopsies might not provide appropriate histologic results because it could change the tumor’s DNA and cellular features into necrotic clumps [2]. Given the unsuccessful biopsies in June 2021 and the abrupt worsening of presenting symptoms within one month, the patient was unlikely to benefit from delaying treatment in an attempt to obtain a better sample. Symptomatic relief with BiPAP, head elevation, steroids, and external radiation provided the patient with the most comfort via the least invasive measures. Radiation therapy has been shown to be 80% effective in providing relief from symptoms of SVC syndrome, though definitive doses and fractions for radiation need to be modified on a per-patient basis [3]. Oncologic radiation doses can differ based on curative or palliative treatment. The total dose, dose per treatment, and an overall number of treatments are not only adjusted based on the malignancy itself, but also on the patient’s end goal. Radiation therapy with curative goals is required to decrease the toxicity to normal tissues [4]. Palliative radiation therapy consists of high-dose radiation and a limited number of sessions. Some research suggests approximately 30 Gy in 10 treatment sessions (3,000 Gy total) is beneficial to patients with lung malignancies, which is what our patient received [4]. This regimen is associated with small improvements in symptom control and survival, but there are increased risks of short-term adverse effects like esophagitis [2].

Percutaneous stenting of the SVC has also been used to diminish symptoms of SVC syndrome. Stenting, when feasible, has shown faster symptom resolution, with subjective symptom resolution within 72 hours post-procedure [1]. This procedure is superior because it does not alter the surrounding tissue; biopsy and histology can still be done after the placement of the stent. Compared to stenting, depending on the radiation regimen, radiation shows a subjective symptom improvement response in three to 30 days [1]. As per our patient's wishes, stenting was invasive and not an option she was receptive to.

The patient's presentation to our ED was an urgent clinical emergency that required a multi-team approach to balance the wishes of the patient with capacity. The family’s support in adhering to the wishes of their mother was a challenge in itself, but it provided the team guidance in offering a route to pursue and actions to take. 

The World Health Organization defines palliative care as an approach that improves the quality of life of patients with life-threatening illnesses, by preventing and relief of suffering while attending to their needs with a holistic approach [2]. Patient rights entail them to autonomy to make informed decisions based on their capacity to understand, clearly express their goal of treatment, and understand their situation and the consequences of their decision to refuse treatment [5]. Our medical team called upon the palliative service to work in tandem with the patient’s family to provide goal-driven treatment reflective of this definition. 

The act of utilizing therapeutic privilege comes to mind in situations such as these where a treatment option (i.e., surgery or stenting) could provide complete resolution of the symptomology and crisis. Thus, the act of informed consent, family discussions, and advance directive supersedes evidence-based therapeutic resolution [6]. Patient autonomy gives patients the right to make an informed decision. Refusal of care falls under the rights patients and families have for their care. A good clinician and a multi-team approach resolve such crises with appropriate follow-through and upholding beneficence and non-maleficence [7].

After 17 days, 10 radiation treatments, and supplemental oxygen via BiPAP, high flow nasal cannula, and regular flow nasal cannula with supportive care, the patient was able to return home for hospice care with supplemental oxygen. Palliative radiation alleviated the patient’s symptoms enough to provide her comfort from SVC syndrome.

## Conclusions

In conclusion, this case represents an oncologic emergency in a patient that was set on refusing invasive diagnostic or therapeutic procedures but still required fast decision-making and treatment to avoid rapid decompensation and alleviate her respiratory distress. This case required our care team to display shared decision-making of the treatment plan with the patient and her family. A palliative approach to treatment with radiation therapy to lessen her subjective symptoms as well as shrink the tumor that was impinging on the SVC was agreed upon as the best approach. It is crucial for clinicians to urgently utilize procedures like palliative radiation when dealing with oncologic emergencies, while balancing the wishes of patients. This patient was elderly, had full capacity, with family support, and did not want to undergo invasive surgeries and procedures, hence exercising her patient rights of autonomy to refuse care on the grounds of appropriate capacity. The emphasis on palliative radiation was appropriate, which in the end allowed her to return home with reduced symptoms aligned with her stated wishes of none invasive therapeutics.
